# Dispelling urban myths about default uncertainty factors in chemical risk assessment – sufficient protection against mixture effects?

**DOI:** 10.1186/1476-069X-12-53

**Published:** 2013-07-01

**Authors:** Olwenn V Martin, Scholze Martin, Kortenkamp Andreas

**Affiliations:** 1 Institute for the Environment, Brunel University, Kingston Lane, Uxbridge UB8 3PH, United Kingdom

**Keywords:** Acceptable daily intake, Extrapolation, Hazard assessment, Mixture, Risk assessment

## Abstract

Assessing the detrimental health effects of chemicals requires the extrapolation of experimental data in animals to human populations. This is achieved by applying a default uncertainty factor of 100 to doses not found to be associated with observable effects in laboratory animals. It is commonly assumed that the toxicokinetic and toxicodynamic sub-components of this default uncertainty factor represent worst-case scenarios and that the multiplication of those components yields conservative estimates of safe levels for humans. It is sometimes claimed that this conservatism also offers adequate protection from mixture effects. By analysing the evolution of uncertainty factors from a historical perspective, we expose that the default factor and its sub-components are intended to represent adequate rather than worst-case scenarios. The intention of using assessment factors for mixture effects was abandoned thirty years ago. It is also often ignored that the conservatism (or otherwise) of uncertainty factors can only be considered in relation to a defined level of protection. A protection equivalent to an effect magnitude of 0.001-0.0001% over background incidence is generally considered acceptable. However, it is impossible to say whether this level of protection is in fact realised with the tolerable doses that are derived by employing uncertainty factors. Accordingly, it is difficult to assess whether uncertainty factors overestimate or underestimate the sensitivity differences in human populations. It is also often not appreciated that the outcome of probabilistic approaches to the multiplication of sub-factors is dependent on the choice of probability distributions. Therefore, the idea that default uncertainty factors are overly conservative worst-case scenarios which can account both for the lack of statistical power in animal experiments and protect against potential mixture effects is ill-founded. We contend that precautionary regulation should provide an incentive to generate better data and recommend adopting a pragmatic, but scientifically better founded approach to mixture risk assessment.

## Background

Predicting the potential detrimental effects on human health of toxicants requires the extrapolation of experimental data in animals to the general population. This is routinely conducted by dividing the highest dose not exhibiting observable toxicity, no observed adverse effect levels (NOAELs), by a default uncertainty factor of 100.

In the years preceding the implementation of the European Community Regulation on the Registration, Evaluation, Authorisation and Restriction of Chemicals (EC 1907/2006) (REACH) in June 2007, a number of extensive reviews of the literature concerning uncertainty factors were published by government agencies and industry associations [1-5]. Recent developments regarding our understanding of the differences in sensitivity to the toxic effects of chemicals between species and between individuals alone render a re-analysis both timely and necessary. This is also now crucial because a number of myths about the supposed properties of uncertainty factors do persist and these might have serious implications for the regulation of mixtures. Principally, it is often assumed that default uncertainty factors represent worst-case scenarios and that the multiplication of their component factors yields sufficiently conservative, or as some would argue, even overly conservative estimates of safe levels of contaminants for humans [6]. As a result, it is also assumed that this degree of conservatism offers adequate protection not only from the toxic effects of individual substances but also from the mixture effects of substances. This idea is expressed in relation to mixtures composed of chemicals that act by different mechanisms of toxicity, but is often thought to apply to mixtures generally, regardless of composition or mode of action. This was recently enunciated by the European Commission Scientific Committees in their opinion on the toxicity and assessment of chemical mixtures [6], and echoed in the recent communication of the European Commission on the combination effects of chemicals [7]. Where mixtures are made up of chemicals thought to have dissimilar modes-of-action, mixture toxicology theory predicts that the mixture will be without effect provided that all its constituents can be shown to have no effect individually. Although the European Commission Committees now recognise that the ‘no observed adverse effect’ levels (NOAELs) derived experimentally do not always represent absolute zero-effect levels due to lack of statistical power, they conclude that conservative assumptions made when deriving safe levels for humans, in other words the application of uncertainty factors, render the possibility of mixture effects unlikely following exposure at the said safe levels.

These ideas are correct if two unspoken assumptions are fulfilled: first, that the diversity of chemicals which make up human exposures act in strictly independent ways, according to the stochastic principles of the mixture concept of independent action [8]. Under these conditions, a combination effect is not expected if all components in the mixture are present at levels equivalent to zero effects. However, cases where experimental mixtures were shown to follow the principles of independent action are few and far between. To date, the applicability of independent action for multi-component mixtures has been established for algae [9] and bacteria [10], but empirical data demonstrating that the concept is valid in mammalians is missing altogether [11]. There is however ample evidence that combination effects in mammals can be adequately described by the alternative concept of dose addition [12]. However, under dose addition, combination effects may occur even when all mixture components are present at levels below dose thresholds associated with zero effects.

In this review we examine the second assumption implicit in the widely held view of human risk assessors, so succinctly expressed by the EU Scientific Committees, namely that current exposures to chemicals generally pose negligible risks, in line with an “intended level of protection”. Considering that human populations come into contact with a multitude of chemicals not appropriately assessed toxicologically, this assumption may be somewhat hard to justify in its generality. However, if we restrict the debate to chemicals for which acceptable or tolerable daily intakes (ADI, TDI) are available, the issue to be examined becomes whether ADI and TDI generally can be assumed to represent negligible risks, or perhaps even zero effects. This would be required to fulfil the condition needed to rule out combination effects under independent action. If this condition is not met, there is a strong case for considering mixture effects as part of chemicals risk assessment.

From the perspective of mixture toxicology, the answer to this question depends on the conservativeness of the uncertainty factors that are used to translate NOAELs into ADIs and TDIs. Are they generally sufficiently protective to safeguard against the possibility of mixture effects?

We deal with this issue by assessing the perceived conservativeness of uncertainty factors through tracing their origins. From a historical perspective up to the present day, we first summarise the evolution of the thinking behind uncertainty factors and the unknown quantities they are purported to represent. In the second part of this paper, the data available to assess the component differences in sensitivity that uncertainty factors are supposed to account for is compared against default values in order to gauge the level of protection afforded. Next, we consider this level of protection with respect to the probabilistic approaches applied to the multiplication of component factors. This will put us in a position to assess the conservativeness of default uncertainty factors used in human risk assessment and standard setting. If they can be shown to be insufficiently protective when dealing with single chemicals, it will be hard to argue that they also protect against the effects of combined exposures.

Finally, we return to mixtures risk assessment with the aim of assessing whether current risk assessment practices implicitly assume that assessment factors take account of combination effects.

### Historical perspective – is the default a worst-case scenario?

The introduction of factors interchangeably referred to as safety, uncertainty, correction, assessment, adjustment or extrapolation factors cannot be separated from the need to derive safe levels of additives or contaminants in food. These factors originate from the emergence of the ADI, a concept widely credited to European and American toxicology experts including Truhaut through his involvement in international agencies such as the World Health Organisation (WHO), Food and Agriculture Organization (FAO), International Union Against Cancer, Council of Europe and the European Committee for the Protection of the Population Against the Hazards of Chronic Toxicity (EUROTOX) in the early 1950s [13]. The idea of an ADI is based on the first tenet of toxicology as enunciated by Paracelsus: namely that “All things are poison, and nothing is without poison; only the dose permits something not to be poisonous.” which is interpreted to mean that there is a dose or threshold below which exposure to a chemical would not result in any detrimental effect (carcinogenic chemicals are considered an exception to this rule).

#### Acceptable daily intake, ‘appreciable risk’ and the desired level of protection

The ADI was first defined in the WHO Technical Report 240 as “the daily dosage of a chemical, which, during an entire lifetime, appears to be without appreciable risk on the basis of all the facts known at the time” [14]. The term ‘without appreciable risk’ has been mirrored in European and American definitions of ADIs or Reference Dose (RfD) respectively [15,16]. A more recent development is the explicit inclusion of sensitive or susceptible subgroups. Although ‘appreciable risk’ has never been quantitatively defined, guidance from the WHO emphasises that “the ADI was an expression of opinion, which carried no guarantee of "absolute" safety” [17]. The most recent guidance from the WHO does however mention that dose–response assessment can be “used to define the dose associated with a negligible (e.g. 1 in a million) increased response over background” [18]. The report produced by a Committee on Improving Risk Analysis Approaches Used by the USEPA also defined a risk-specific reference dose (for quantal effects) as “the dose that corresponds to a particular risk specified to be *de minimis* (for example, 1 in 100,000) at a defined confidence level (for example, 95%) for the toxicity end point of concern.” National Research Council, 2008 [16], giving some tacit indications of both the order of magnitude of risk (0.001-0.0001% of the population) that would be considered appreciable as well as the associated uncertainty. This appears to endorse Hattis et al [19]. ‘Straw Man’ proposal for a quantitative definition of the RfD, namely:

● “The daily dose rate that is expected (with 95% confidence) to produce less than 1/100,000 incidence over background of a minimally adverse response in a standard general population of mixed ages and genders, or

● “The daily dose rate that is expected (with 95% confidence) to produce less than 1/1,000 incidence over background of a minimally adverse response in a definable sensitive subpopulation.”

While there are no legally binding quantitative definitions of desired level of protection for non-carcinogenic chemicals, the ‘Straw Man’ proposal has come under debate. There are experimental, statistical and mathematical limitations to deriving risk estimates at the level of 1 in 100,000 additional cases with any meaningful degree of certainty. It is clear that regardless of its exact value, the desired level of protection will have to be extrapolated from the observed data (animal experiments) by several orders of magnitude. Many non-threshold dose–response models fit the toxicity data equally well but differ vastly in terms of effect estimates at low doses.

A detailed discussion of low level effects is beyond the scope of this review. It should nonetheless be noted that it is now widely recognised that even if the existence of biological zero-effect levels is assumed, these cannot be quantified, neither at an individual nor at a population level [20]. Although often misunderstood, NOAELs cannot be equated with zero-effect levels. They are also not “natural constants” as their numerical value is strongly influenced by the experimental design, such as the spacing of doses, number of animals and the sensitivity of the endpoint considered. In general, fewer tested doses and lack of statistical power will produce higher NOAELs. In some cases, NOAELs are associated with effect magnitudes as high as 25% (see the discussion in [21]). It has also long been recognised that the degree of protection achieved varies not only with the size of the factor but also the slope of the log dose–response curve [22,23]. The benchmark dose level (BMDL) has been proposed as an alternative to the traditional NOAEL approach [24]. The BMDL is defined as the lower statistical confidence limit of the dose resulting in a predetermined response. It has attracted interest because it accounts for the shape of the dose–response curve and the quality of study design, thereby providing an incentive to conduct better studies, and it is not restricted to doses tested experimentally [25]. It has been proposed that linear extrapolation from an observed point of departure such as a NOAEL or BMDL may be the least uncertain method of assigning reasonable upper bound estimates of risk for both cancer and non-cancer endpoints [26,27].

In summary, although there is no legally binding quantitative definition of ‘appreciable risk’ or the desired level of protection, it is generally accepted that it ought to be of a magnitude equivalent to 0.001-0.0001% over background incidence, in line with Hattis’ “Straw Man proposal”. Difficulties arise, however, as different methods of deriving the dose corresponding to this response may themselves yield estimates that differ by several orders of magnitude.

#### Safety factors and adequate margin of safety

Because it would not be ethically acceptable to test toxicity on human populations, a safety factor of 100 was originally proposed by Lehman and Fitzhugh [28] of the US Food and Drug Administration. To derive a level considered safe for humans, the safety factor was combined with a NOAEL established in chronic animal studies after oral administration of the substance of interest. A safety factor of 100 was arbitrarily set, but was originally assumed to cover interspecies (animal-to-human) variability, and inter-individual (human-to-human) variability, which allowed sensitive human populations to be compared with healthy experimental animals. It was also assumed to protect against possible synergistic action of the many xenobiotics found in food. In June 1957, the Joint FAO/WHO Expert Committee on Food Additives (JECFA) met in Geneva to discuss the issue. The Committee stated that “in the absence of any evidence to the contrary” an arbitrary factor of 100 applied to the maximum ineffective dose in animals calculated in mg/kg bodyweight provided an adequate margin of safety [29]. This margin of safety was purported to allow for “any species difference in susceptibility, the numerical differences between the test animals and the human population exposed to the hazard, the wider variety of complicating disease processes in the human population, the difficulty of estimating the human intake and the possibility of synergistic action among food additives” [29].

### Mixture effects

Sometime between the 1970s and the early 1980s, the consideration of possible mixture effects within an overall safety factor faded in favour of a division of the 100 factor into two sub-factors of 10, each accounting for either intra- or interspecies variability [30-32]. Even though it was recognised that additive or synergistic effects needed to be considered in the derivation of an ADI, the Joint FAO/WHO Meeting on Pesticide Residues (JMPR) concluded in 1981 that although there was a need for further data on interactions of pesticides, the consideration of mixtures did not require any change in the general principles for estimating ADIs. The rationale for this decision was given by the following justifications [17]:

● “Not only could pesticides interact, but so could all compounds (including those in food) to which man could be exposed. This leads to unlimited possibilities, and there is no special reason why the interactions of pesticide residues (which are at very low levels) should be highlighted as being of particular concern;

● “Very little data on these interactions are available;

● “The data obtained from acute potentiation studies are of little value in assessing ADIs for man.”

It is interesting to note that not the purported intrinsic conservativeness of safety factors, but the complexity of the issue and a lack of data were chosen as justifications for discounting mixture effects. If the default overall safety factor of 100 was originally intended to account for mixture effects, this intention has been abandoned thirty years ago.

#### Towards chemical-specific adjustment factors

The International Programme on Chemical Safety (IPCS) stated that the safety factor “is intended to provide an adequate margin of safety for the consumer by assuming that the human being is 10 times more sensitive than the test animal and that the difference of sensitivity within the human population is within a 10-fold range” [33]. This statement effectively spells out hypotheses that have subsequently been both subject to disputed interpretations and have stimulated research efforts intended to confirm its scientific basis. For environmental chemicals, a number of additional factors were proposed to account for various shortcomings in the experimental data being extrapolated to a Tolerable Daily Intake (TDI) or RfD, such as inappropriate design, using acute or subchronic rather than chronic data, or a lowest observable adverse effect level (LOAEL) rather than a NOAEL, as well as other factors such as severe or irreversible effects [22,34-36].

The further subdivision of the conventional 100-fold safety factor according to two aspects of toxicity, toxicokinetics and toxicodynamics, was elaborated by Renwick [36,37]. Renwick’s preliminary analysis of a few available toxicokinetic and toxicodynamic differences between species and within the human population for a few pharmaceuticals and artificial sweeteners led him to consider the default 10 × 10 factors not as a ‘worst-case’ but rather as an ‘adequate’ scenario. Overall safety factors between 1.2 and 7,500 were tentatively drawn and a factor approaching 10,000 was suggested as more appropriate when a particular subset of the population is thought to be particularly sensitive [37]. In a subsequent article from 1993 where the rationale for the subdivision of each 10 sub-factor was elaborated, Renwick admitted that the limited data presented would support applying a 10-fold factor to interspecies toxicokinetics alone [36]. He justified the introduction of a 4-fold factor for inter-individual kinetic differences with reference to the observation that in healthy adults the coefficient of variation in kinetic parameters for an artificial sweetener and six pharmaceutical compounds was 80% or less for 5 of the 7 compounds. If a normal (Gaussian) distribution were assumed to underlie these values, the 99th percentile for a standard deviation of 80% would correspond to 2.9 times the mean value. The value of 4 was selected on the basis of the limited size of the individual studies and was supported by a more extensive review published by Hattis et al. [38]. It was nonetheless recognised that inter-individual differences in kinetics alone may greatly exceed a 10-fold span.

**Figure 1:**
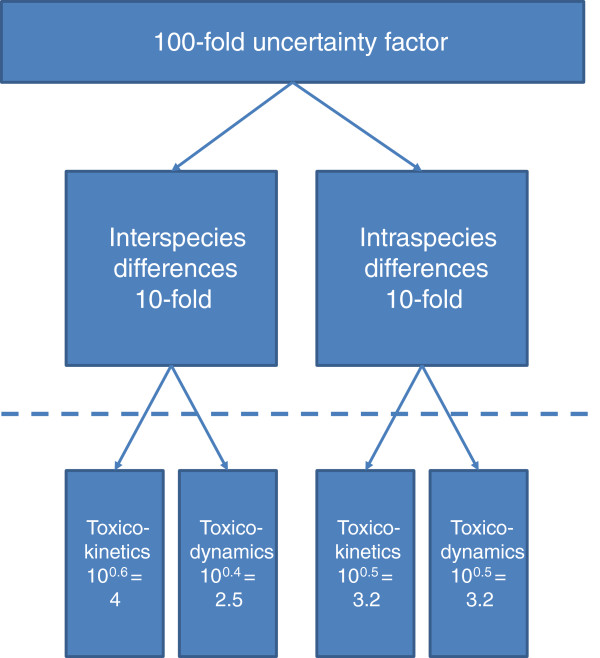
Subdivision of the 100-fold default uncertainty factor adopted by IPCS.

With regards to inter-individual toxicodynamic differences, the default value of 2.5 for the sub-factor was based on acute effects of pharmaceutical compounds in healthy adults. Although data would have supported a higher value, the default value was justified on the basis that “inter-subject variability in specific drug receptor sensitivity would be greater than less specific toxicological phenomena” [36]. Although it was recognised that these values were based on data obtained in healthy adults and may therefore not apply to particularly sensitive groups such as the elderly and the diseased, it was recommended that specific health advice be given to potentially “at risk” minorities, considering exposure to food additives and contaminants. Further, Renwick [36] concluded that default values were chosen to fit the 100-fold overall factor such that adoption of the proposed scheme “would not require a cataclysmic re-evaluation of all possible toxicants!”. In 1994, the scheme was adopted in modified form by IPCS. IPCS considered toxicokinetic and toxicodynamic inter-individual differences were similar and the 10-fold factor inter-individual variability was equally sub-divided between the two aspects (10^0.5^ = 3.2) as illustrated in Figure 1[39].

The sub-division of the default 100-factors was championed to allow the replacement of individual default sub-factors by values derived from chemical specific data when available, and guidance for the use of chemical or pathway-specific adjustments was subsequently drawn by the IPCS [40]. The use of toxicokinetic and toxicodynamic preclinical and clinical data in humans has been investigated for setting occupational exposure limits for pharmaceutically active ingredients [41-43], as well as natural compounds as probe substrates for the cytochrome P450 (CYP) 1A2 to derive a pathway-specific kinetic adjustment factor for human variability [44] (CYPs are a large and diverse group of enzymes involved in the metabolic oxidation of xenobiotics). These studies recognised that the existence of two or more distinct genetic forms (polymorphisms) of enzymes involved in xenobiotic metabolism could give rise to multimodal sensitivity distributions for human populations. In such cases, the inter-individual toxicokinetic variability sub-factor should be equal to the ratio of the furthermost tail of the distribution (protecting 95 or 99% of the sensitive subpopulation) and the median for normal healthy individuals. Whilst conceding that the default toxicokinetic sub-factor for human variability (3.2) would not protect sensitive subgroups such as neonates from chemicals metabolised by CYP 1A2, Renwick et al. [44] nevertheless recommended that a pathway-specific factor be derived for healthy young adults, and sensitive subgroups protected by risk management measures. Alternatively, he suggested that a constant proportion of the combined population be used to derive an uncertainty or adjustment factors in order to protect sensitive subgroups which cannot be recognised by risk managers (as is the case with polymorphisms, where most people would not be aware of their specific genetic susceptibility). Nonetheless, it is clear that the use of chemical or pathway-specific adjustment factors, instead of a default uncertainty factor, could yield greater as well as smaller overall adjustment factors. In summary, the default values for the overall uncertainty factor and its sub-components do not represent a worst-case scenario, and were never intended to do so.

### Current European guidance

The scientific validity of the default values of assessment factors and sub-factors has been the subject of several comprehensive reviews in the peer-reviewed literature as well as reports in the grey literature. In the default values recommended by the European Chemicals Agency (ECHA) for use under the REACH Regulation are reproduced together with values recommended by various other agencies [45]. The recommendations of the Swedish National Chemicals Inspectorate (KEMI) were not included in the annex of the ECHA guidance but are presented here. As the focus of this work is on oral exposure, a factor that can be applied to extrapolate between different routes of exposure has been omitted from this table. It is interesting to note at this stage that ECHA recommends using an allometric scaling factor in combination with a sub-factor of 2.5 for toxicodynamics to account for interspecies differences. As a result, the interspecies subfactor may be greater or smaller than 10 depending on the experimental animal species from which a safe level is derived. KEMI and ECHA recommend the use of an extra factor of up to 10 in cases where the database from which a safe level in experimental animals is selected is considered to not appropriately assess potential effects on children [4,45] Table 1.

**Table 1:** Summary of default assessment factors used in human health risk assessment (adapted from ECHA 2008)

**Assessment factors**	**WHO-IPCS **[17][22][33][39]	**USEPA **[46]	**ECETOC **[1]	**Danish EPA **[47]	**UBA/BAuA **[3][48]	**TNO/RIVM **[5][49]	**KEMI **[4]	**ECHA Guidance **[45]
Database adequacy	1-10			1-100		1	1-5	1
LOAEL to NOAEL	3-10	10	3		10	1-10	3-10	3
Severity of Effect	1-10					1	1-10	
Duration of exposure		10						
Sub-chronic to chronic			2		2-3	10	3	2
Sub-acute to sub-chronic					2–3	10	8	3
Sub-acute to chronic			6		6-7	50-100	24	6
Interspecies	10	10		10		10	10	
Toxicokinetics	4.0						4	
Allometric scaling								
Mouse			7		7			7
Rat			4		4			4
Monkey			2		2 or 4			2
Dog			2		2			1.4
toxicodynamics	2.5				2-3		2.5	2.5
Intraspecies	10	10	5	10	25	10	10-16	10
Toxicokinetics	3.16				8		3–5	
Toxicodynamics	3.16				3		3.16	
Modifying factor		≤10						

In this chronological perspective we were able to establish that the default uncertainty factor is no longer intended to account for mixture effects, nor does it represent a worst-case scenario. However, later we will see that it still holds sway in some unspoken assumptions about the degree of protection afforded by these factors. It is therefore interesting to critically review and update the data used to justify the values for interspecies and intraspecies or interindividual variability in the reports summarised in Table 1. This is important in order to gauge the degree of conservatism afforded by the default 100 assessment factor. Component subfactors are considered individually first, before attention is given to their combination.

### Interspecies variability

#### Allometric scaling

Interspecies differences in responses to toxic chemicals may arise from differences in body size alone. This is usually accounted for by scaling doses to body weight. Accordingly, NOAEL, LOAEL and BMDL as well as the safe levels derived from these estimates are expressed in mg/kg body weight/day. During the development of the ADI concept, the dose metric of mg/body surface area was also considered, and although its scientific merits were recognised, the familiar metric of mg/kg body weight was adopted for practical convenience [13,50]. Many physiological functions correlate better with body surface or caloric demand than with body weight. An allometric equation raising the body weight to the power 0.75 or 0.67 for caloric demand or surface area, respectively, can be used to calculate species-specific allometric scaling factors for application to doses expressed in mg/kg body weight. For common test species, estimates for these factors are given in Table 2. Kalberlah and Schneider [3] reviewed efforts to derive the allometric exponent, and this yielded values between 0.42 and 0.97. They also found that approaches using caloric demand or surface area largely produce similar results. On theoretical grounds, extrapolations based on caloric demand are generally preferred to those based on body surface [49]; clearance rates of chemicals and other time-dependent parameters as well as the consumption of nutrients appear to correlate with body weight raised to the power of three quarters [51]. Watanabe et al. [52] reanalysed data used to derive coefficients of the allometric equation and found that scaling according to caloric demand provided a better fit. In view of the variance in the data, they nevertheless advocated scaling according to the more precautionary surface area [21,52]. It should be stressed that these investigations were carried out considering the acute toxicity of antitumour drugs. Agents such as these interfere with growth processes and are expected to follow more closely allometric scaling, but this may not hold true for toxicants with other modes of action [51]. An extensive review of drug metabolism suggested that if renal disposition correlates adequately with allometric scaling, this may not be the case for hepatic metabolism [53]. It has therefore been recommended that an additional factor be applied to data from experimental animals to extrapolate safe levels in humans, in addition to dose extrapolation across species according to allometry [54].

**Table 2:** Allometric scaling factors according to caloric demand and surface area for common experimental species

**Test species**	**Scaling factor for caloric demand**	**Scaling factor for surface area**
Mice	7	14
Rats	4	6
Rabbits	2.4	3
Dogs	1.4	1.7

#### Deviations from allometric rules

Brain weight and oxygen consumption also diverge from allometric scaling and this may mean that humans are more sensitive to neurotoxic effects than experimental animals. Another interesting example of deviation from the allometric rules is that of polychlorinated biphenyls (PCBs) for which NOAEL values for rats, mice and monkeys differed by several orders of magnitude [55]. Reproductive endpoints also depart from the allometric principle. Compared to rodents, the male human has relatively low sperm numbers and numbers of motile sperm with respect to that required for fertility [3].

In addition to interspecies variations that can be explained in terms of differences in size alone, there are differences in susceptibility to toxic injury related to species-specific toxicokinetic and toxicodynamic factors.

### Toxicokinetic interspecies differences

Schneider et al. [56] compared pharmacokinetic parameters for the mouse, rat, guinea pig, hamster, cat, rabbit, dog, monkeys and the human. Their literature search yielded data for 71 substances and they calculated species ratios that were in good agreement with allometric scaling according to caloric demand. Griem et al. [57] carried out an extensive review of *in vitro* data on the enzymatic transformation of substances in the rat, mouse and human for the liver, respiratory tract kidney and gastrointestinal tract. Some differences in enzymatic transformation of xenobiotics were observed between experimental animals and humans. These differences between species were not found to be systematically higher or lower for a given enzyme but rather enzymatic activity differed according to the organ system considered. For example, there were no discernible differences in *in vivo* CYP activity between rodents and humans, whereas *in vitro* CYP activity was considerably higher in the lungs of rodents compared to humans. An empirical model was used to assess the relevance of species differences observed in the liver *in vitro* for *in vivo* metabolism. Species differences observed *in vitro* were accurately predicted to persist *in vivo* for enzyme-substrate complexes with a low intrinsic clearance (the ability of organs such as the liver and kidney to metabolise or excrete a substrate). For higher intrinsic clearance rates (approximately five times the hepatic blood flow or higher), substrate transport in the blood became the rate-determining factor and interspecies ratios of hepatic clearance were in agreement with allometric scaling according to metabolic rate. Walton et al. [58-60] carried out literature searches of pharmacokinetic and metabolism studies in humans and animals for probe substrates of CYP1A2, glucuronidation and for compounds excreted unchanged in urine. Mean clearance ratios following oral absorption of CYP1A2 substrates were 6.2 and 10.2 for the rat and the mouse, respectively, compared to the human, evidently larger than the 4 default value assigned to the subfactor for toxicokinetic interspecies differences or the allometric scaling factors for those species [58]. The review of compounds excreted as glucuronides uncovered important differences between humans and test species, with some compounds excreted unchanged in test species, but not in humans. Another significant factor that would influence the internal dose was the extent of enterohepatic recirculation. The ratios of clearance values (the dose divided by the total systemic dose) in humans compared to test animals for drugs that were glucuronidated in humans varied from 38 for oxazepam in the rat to 0.095 for zomepirac in the rabbit [59]. For compounds excreted unchanged primarily by renal excretion in both the human and experimental animals (following intravenous injection), ratios of plasma clearance in animals compared to humans varied from 0.26 in the dog for 1-aminocyclopropanecarboxylic acid to 35 in the mouse for cefotetan. The extent of plasma protein binding has a marked influence on tissue distribution and rates of excretion particularly by glomerular filtration [60]. Toxicity also depends on the active form of the chemical, depending on whether toxicity is due to the parent compound, a highly reactive metabolite which disappears rapidly, or a stable metabolite capable of entering the circulation. Clewell et al. [61] used pharmacologically based pharmacokinetic models where they applied mouse, rat and human parameters. They found that interspecies differences in toxicokinetics alone could exceed the default interspecies factor of 10 by up to ten-fold depending on the toxic form of the chemical.

### Toxicodynamic interspecies differences

There is generally a paucity of data regarding differences of sensitivity between species due to toxicodynamic parameters. A well known example of such variation is that of the acute toxicity of 2,3,7,8-tetrachlorodibenzo-p-dioxin (TCDD); differences of up to four orders of magnitude in the median lethal dose (LD50) between hamsters and guinea pigs were attributed to varying concentrations and functions of the intracellular arylhydrocarbon receptor (AhR) [3].

There is therefore evidence that the sensitivity of experimental animals to the toxicity of chemical substances can deviate from allometric scaling and that there are also toxicokinetic and toxicodynamic differences between animals and humans that may render animals either more or less sensitive than humans. To assess how frequently, or for which proportion of chemicals an interspecies extrapolation factor may exceed the default values it is necessary to analyse datasets for groups of chemicals rather than individual substances or specific pathways. The following sections review the magnitude of interspecies differences derived from such datasets.

### Quantitative evaluation of interspecies differences

Two types of data have been used for the quantitative evaluation of interspecies differences. Firstly, there are preclinical data for pharmaceuticals in test animals and these have been used to compare toxic doses with those derived from human clinical data (summarised in Table 3). Secondly, comparisons of toxicity evaluations between animal species have been made and these are presented in Table 4.

**Table 3:** Quantitative evaluation on interspecies differences using human and animal data

**Substances dataset**	**Dose metric**	**Species**	**Results**	**Reference**
Poisonous metal compounds	Toxic doses	Rat	2.5<Toxic dose_Rat_/Toxic dose_Human_<152	[31]
			Geometric mean (GM) = **12**	
18 chemotherapeutical agents	Lethal Dose (LD)10 and Maximum Tolerated Dose (MTD)	Mouse, rat, hamster, dog, monkey	LD10 (mg/m^2^)/3 carries 5.9% probability of exceeding MTD_Human_	[62,63]
10 pesticides (single dose) and 12 pesticides (repeated doses)	Various toxic doses	Rat, dog, pig, calf, cow, horse, sheep, steer	1.9<Acute non fatal dose_Animal_/Acute non fatal dose_Human_<100	[31,64]
			GM = **11**.	
			0.58 <Chronic dose_Animal_/Chronic dose_Human_<9.4	
			GM = **2.9**.	
40 anticancer agents	MTD	Dogs, monkeys	MTD/10 carries a clinical risk human toxicity of 3% (3 out of every 100 drugs)	[65]
107 pharmaceuticals (?)	Toxicity indices	4 to 6 laboratory animal species	Extrapolation of doses (mg/kg) from white rats to man exaggerated tolerable dose 5.5- to 40-fold	[66]
25 chemotherapeutic agents	LD10 and MTD	Mouse, rat, hamster, dog, monkey	The predicted MTD_Human_ is overestimated for 20% of agents using 0.75 power (caloric demand) scaling, less with body surface scaling	[52]
26 pharmaceuticals	Maximum therapeutic equivalent dose and toxicokinetic parameters	Up to 4 laboratory animal species	Scaling according to metabolic rate underestimated risk to man in 59% of cases	[67]
63 anti-neoplastic drugs	LD10, Toxic Dose Low (TDL) and MTD	Rat, mouse, hamster, dog, monkey	LD10 or TDL_Mouse_/MTD_human_ = **8.0** (P95 = 50.9)	[68]
			LD10 or TDL_Hamster_/MTD_human_ = **7.6** (P95 = 52.1)	
			LD10 or TDL_Rat_/MTD_human_ = **2.6** (P95 = 21.7)	
			LD10 or TDL_Monkey_/MTD_human_ = **2.4** (P95 = 15.3)	
			LD10 or TDL_Dog_/MTD_human_ = **1.2** (P95 = 7.0)	
61 anti-neoplastic drugs	LD10 and MTD	Mouse, rat, dog, monkey	LD10_Mouse_ /MTD_Human_ >10 for 37% compounds	[69]
			LD10_Rat_ /MTD_Human_ >10 for 19% compounds	
			LD10_Monkey_ /MTD_Human_ >10 for 5% compounds	
			LD10_Dog_/MTD_Human_ >10 for 3% compounds	

**Table 4:** Quantitative evaluation on interspecies differences using only animal data

**Substances dataset**	**Dose metric**	**Species**	**Results**	**Reference**
190 chemicals	TD50	Mouse and rat	Median TD50_Mouse_/TD50_Rat_ ratio = 2.4	[70]
			20% of ratios exceeded 10	
69 pesticides	NOAEL	Mouse, rat and dog	NOAEL_Rat_ /NOAEL_Dog_ : 1.58 (0.99-2.24)	[71]
			NOAEL_Mouse_ /NOAEL_Rat_ : 3.87 (2.24-6.32)	
			NOAEL_Mouse_ /NOAEL_Dog_ : 7.07 (3.53-13.42)	
Binary interspecies comparisons from dozens to over 500 agents	Mostly LC50	Aquatic species	Orders-within-Classes Extrapolation	[72]
			Weighted mean of uncertainty factor (UF) prediction interval (PI): 26 (95% PI), 35 (99% PI)	
			Upper 95% UFs: 65 (95% PI), 88 (99% PI)	
184 substances	NOAEL	Mouse, rat, dog	NOAEL_Rat_ /NOAEL_Dog_ : 1.3 (P95 =18.8)	[49]
			NOAEL_Mouse_ /NOAEL_Rat_ : 4.2 (P95 = 73.9)	
			NOAEL_Mouse_ / NOAEL_Dog_ : 6.4 (P95 = 124.6)	
198 substances	NOAEL	Mouse, rat, dog	NOAEL_Rat_ /NOAEL_Dog_ : 2.3 (P95 =27)	[73]
			NOAEL_Mouse_ /NOAEL_Rat_ : 3.2 (P95 = 37)	
			NOAEL_Mouse_ /NOAEL_Dog_ : 5.9 (P95 = 50)	
217 substances	LD50	Mouse, hamster, guinea pig, rat, cat, rabbit, monkey dog	LD50_Mouse_/LD50_Rat_= 0.86 (P95 = 2.52)	
			LD50_Hamster_/LD50_Rat_= 1.2 (P95 = 1.33)	
			LD50_Guinea pig_/LD50_Rat_= 0.71	
			(P95 = 2.96)	[68]
			LD50_Cat_/LD50_Rat_= 0.31 (P95 = 1.47)	
			LD50_Rabbit_/LD50_Rat_= 0.76 (P95 = 2.46)	
			LD50_Monkey_/LD50_Rat_= 0.63 (P95 = 0.88)	
			LD50_Dog_/LD50_Rat_= 0.82 (P95 = 4.87)	
216 pesticides	NOAEL	Mouse, rat, dog	NOAEL_Mouse_/NOAEL_Rat_ = 2.22 (P95 = 24.2) NOAEL_Rat_/NOAEL_Dog_ = 1.70 (P95 = 16.6)	[68]
			NOAEL_Mouse_/NOAEL_Dog_ = 6.00 (P95 = 44.8)	
58 compounds	NOAEL and Critical Effect Dose (CED)	Mouse, rat	NOAEL_Mouse_/NOAEL_Rat_ : P95 = 15.5; GSD = 3.4	[74]
			CED_Mouse_ /CED_Rat_ : P95 = 5.6	
			Default value of 10 corresponds to 49th percentile and 71st percentile of mouse to human and rat to human distributions respectively	

Comparisons of toxic or tolerable doses between animals and humans have been conducted mostly for anti-neoplastic agents. There is considerable overlap between the datasets used for these analyses. Further, this type of data suffers from a number of limitations that were best enunciated by Price et al. [69];

● Maximum tolerated doses (MTD) for anti-cancer drugs in humans are by definition associated with toxic effects in some subjects, and therefore cannot easily be compared with NOAELs derived from animal experiments where effects are usually less obvious.

● Data were drawn from short-term studies of acute toxicity rather than chronic exposure.

● The toxicological endpoints used across species or across compounds may differ.

● Anti-cancer drugs are normally administered by injection and do not reflect interspecies differences in absorption from the gastro-intestinal tract or first-pass metabolism in the liver.

● Many anti-neoplastic agents are direct-acting compounds that target rapidly dividing cells and are therefore not representative of the universe of general chemicals.

● MTDs for chemotherapeutic drugs in humans are derived from patients with advanced stages of cancer who are likely to be more sensitive to toxic effects than healthy adults.

● Dose metrics such as MTDs and NOAELs include measurement error.

In addition to chemotherapeutic drugs, a couple of studies analysed data for other pharmaceuticals and Dourson and Stara [31] included two other studies, one reporting values for the toxicity of poisonous metal compounds and another listing data on the toxicity of a limited number of pesticides. Two main conclusions can be drawn from the data presented in Table 3; smaller animals appear less sensitive than larger animals (medians for ratios are generally in good agreement with allometric principles according to basal metabolic rate), and the default value of 10 is likely to be exceeded for a significant proportion of chemicals. The reported values for the 95th percentiles for the distribution of interspecies ratios indicate that variations between chemicals are far greater than interspecies variations.

The rationale for considering differences in toxic responses between animal species is that their magnitude is expected to be similar to the differences observed between animals and humans. The main advantage of this type of dataset is that they include several classes of chemicals and are therefore more representative of the universe of chemicals. Table 4 illustrates again good agreement between measures of central tendency (mean or median) with allometric scaling, whilst measures of spread such as range or percentile values demonstrate large differences in the sensitivity of animal species from chemical to chemical. Chemical-to-chemical variation also appears to be heavily influenced by the endpoints used as a basis for comparisons. Potency differences based on LD50 were much smaller than the species-species differences based on NOAELs. Calabrese and Baldwin [72] applied a different approach to derive an interspecies uncertainty factor of 26 (Table 4). They assumed that susceptibility to toxic substances has an evolutionary basis and is related to phylogenetic distance and used a large aquatic toxicity database, without adjusting for allometry.

In summary, the level of conservatism afforded by the default factor of 10 for interspecies differences depends on the animal species that is considered for analysis. The allometric scaling factor agrees reasonably well with the median of all chemical-specific interspecies factors. Thus, the allometric scaling factor will overestimate interspecies differences for half of the chemicals and underestimate it for the other half. For rodent species routinely used in chemical hazard assessment, the allometric scaling factors (Table 2) are relatively close to 10, and this value may be exceeded for a sizeable proportion of chemicals.

### Intraspecies variability

Although not usually inbred, strains of laboratory animals are genetically fairly uniform and are bred to produce responses with small variations. In contrast, considerably larger inter-individual variation occurs within the human general population. Genetic heterogeneity, age, gender and acquired susceptibility factors such as disease state, diet, stress and previous exposures are among the factors that will lead to marked differences in susceptibility to toxic injury. The scientific basis for the intraspecies default factor of 10 has been scrutinised either by considering variations within the healthy adult population or by considering the sensitivity of specific subsets of the population thought to be particularly vulnerable to toxicity. Here, datasets and studies examining inter-individual variability within the healthy adult population are considered first, before differences in sensitivity related to genetic polymorphisms in metabolising enzymes, due to gender, disease or old age, and finally toxicokinetics in young children are reviewed.

### Inter-individual variability in healthy adults

The only study investigating the overall inter-individual variability of healthy adults is actually based on rats rather than humans. Dourson and Stara [31] used a data set from Weil [75] with dose–response slopes for 490 acute lethality tests of carcinogenic agents in rats to assess the protection afforded by an intraspecies factor of 10. The resulting distribution of adjustment factors (required to scale down a median response by three probits to correspond to 0.13% mortality) suggested that for 8% of the chemicals considered an adjustment factor greater than 10 would be required. The authors acknowledged several limitations to their approach, particularly the use of animal data and the use of LD50s rather than NOAELs. Other analyses of data related to inter-individual differences have considered toxicokinetic variation and toxicodynamic variation separately.

#### Inter-individual toxicokinetic variation

Available datasets related to toxicokinetic parameters in humans are generally restricted to pharmaceuticals, occupational exposures, and some food additives. An early dataset often referred to is that of Hattis et al. [38]. Studies (mostly from 1979–1985) which contained individual human data on pharmacokinetic parameters in at least five healthy adults for 49 chemicals (pharmaceuticals) were retrieved from the scientific literature. The median and 95% range of all geometric standard deviations (GSDs) were reported for each toxicokinetic parameter in two subsequent papers [76,77]. While the medians indicate that the default value of 3.2 for inter-individual toxicokinetics (Figure 1) would be sufficient for healthy adults for 50% of chemicals, the upper limit value of the 95% ranges suggest this may not be the case for all chemicals.

Renwick and Lazarus [78] carried out a similar review and found toxicokinetic data for 60 compounds. If one assumes that toxicokinetic inter-individual variability is log-normally distributed, about 9,000 persons per million would not be protected by the default value of 3.2.

Both datasets were combined and extended and the combined database is freely available (http://www2.clarku.edu/faculty/dhattis/#Web Site Description). It includes data from the peer-reviewed scientific literature as well as some supplied by the US EPA [79]. Figure six in that paper again shows that if the medians of Log_10_(GSD) indicate that the default value of 3.2 would be sufficient for half of all chemicals, their 10-90% range suggest it would not be sufficient when all chemicals are considered.

In an effort to derive uncertainty factors for specific metabolic pathways, Dorne et al. [80] presented the results of several literature reviews of human variability in 14 of the main routes of metabolism including phase I enzymes, phase II enzymes and renal excretion. Ranging from 1.6 to 2.7, the pathway-specific uncertainty factors that protect 99% of the healthy adult population were all smaller than 3.2, when monomorphic pathways, where a gene for a specific enzyme is found in only one form throughout the human species, are considered. However, this was not the case for polymorphic pathways and genetic polymorphisms are discussed as part of sensitive subgroups.

#### Inter-individual toxicodynamic variation

This subfactor (3.2) is intended to allow for inter-individual differences in response to the active form of the compound in the systemic circulation. Data supporting toxicodynamic variability therefore requires separation from kinetic variability. A particular difficulty in ascertaining toxicodynamic variation experimentally is that it would ideally require precise measures of the dose delivered at the target site and this is more readily achieved *in vitro*, *ex vivo* or by pharmacologically based pharmacokinetic (PBPK) modelling. Nonetheless, the earliest dataset on toxicodynamic variability was assembled by Renwick and Lazarus [78] who identified *in vivo* plasma concentration-response data for 49 compounds. Much of the data was for the clinical treatment of patients and disease and aging processes may have contributed to variability. The estimated number of people not covered by the 3.2 default value for inter-individual toxicodynamic variation approached 19,000 per million assuming a lognormal distribution, well above a desired level of protection of 1 per 100,000.

The database described above also contains observations of systemic pharmacodynamic variability for 13 chemicals (Table VI in Hattis et al. [79]). Many of the Log_10_(GSD) (13 out of 21 toxicodynamic parameters) and most of their 5-95% range (19 of 21 toxicodynamic parameters) exceeded 0.2 suggesting that a factor greater than the default value of 3.2 would be needed to account for toxicodynamic intraspecies variability. An update of this database included 41 data groups (measurement of a particular parameter for a particular chemical) relating a response or physiological parameter to the internal concentration after systemic delivery [81]. The 90th percentiles of Log_10_(GSD) of these data was approximately 0.6 suggesting that a 10-fold factor may be justified for pharmacodynamic variability alone in some cases. The authors found that pharmacodynamic variability is generally larger than pharmacokinetic variability.

#### Degree of protection from the intraspecies variation default factor

From both the databases described in the previous sections, the proportion of healthy adults that fall into the range of 10 for inter-individual variability was estimated. Assuming that inter-individual variability is log-normally distributed, Renwick and Lazarus [78] expected that 162 people per million would not be covered by the default uncertainty factor.

From their database, Hattis et al. [82] estimated an overall inter-individual variability intended to cover several types of exposures (e.g. inhalation, ingestion) and effects (chronic or acute). The authors estimated the fraction of people that might show a response at one tenth of the dose that produces a response in 5% of exposed people. The Hattis database also includes some variability in uptake that arguably ought to be accounted for in exposure assessment rather than hazard assessment and the derivation of safe levels. For half the chemicals, nearly 8 people in 100,000 would respond to chronic oral exposure based on perfect drug compliance or about 2 people in 10,000 based on chronic ingestion of a toxicant (includes variability in ingestion behaviour). When considering 95% of chemicals, these values increase to 2 and 3 in every 1,000 people respectively.

It therefore becomes apparent that the default uncertainty factor for intraspecies variability falls short of the level of protection aspired to in the ‘Straw Man Proposal’ (less than 1/100,000 incidence over background with 95% confidence of a minimally adverse response in a standard general population of mixed ages and genders) even when considering exclusively the variability in healthy adults [19]. Further, Hattis et al. [82] enunciate a number of caveats and limitations with the database and methods to quantify variability;

● The toxicity database is assumed to be representative of the universe of chemicals.

● Toxicological parameters for susceptibility are assumed to follow a unimodal Log-normal distribution, however it may not be the case, e.g. when subgroups of the population are particularly sensitive.

● Multiplicative approaches also assume independence of each factor contributing to overall variability. Interdependence of various parameters cannot be excluded and could significantly modify expected fraction of people at the extreme tails of population distribution.

● Distributions of variability also include variability due to measurement error.

### Inter-individual variability in susceptible subgroups

**Figure 2:**
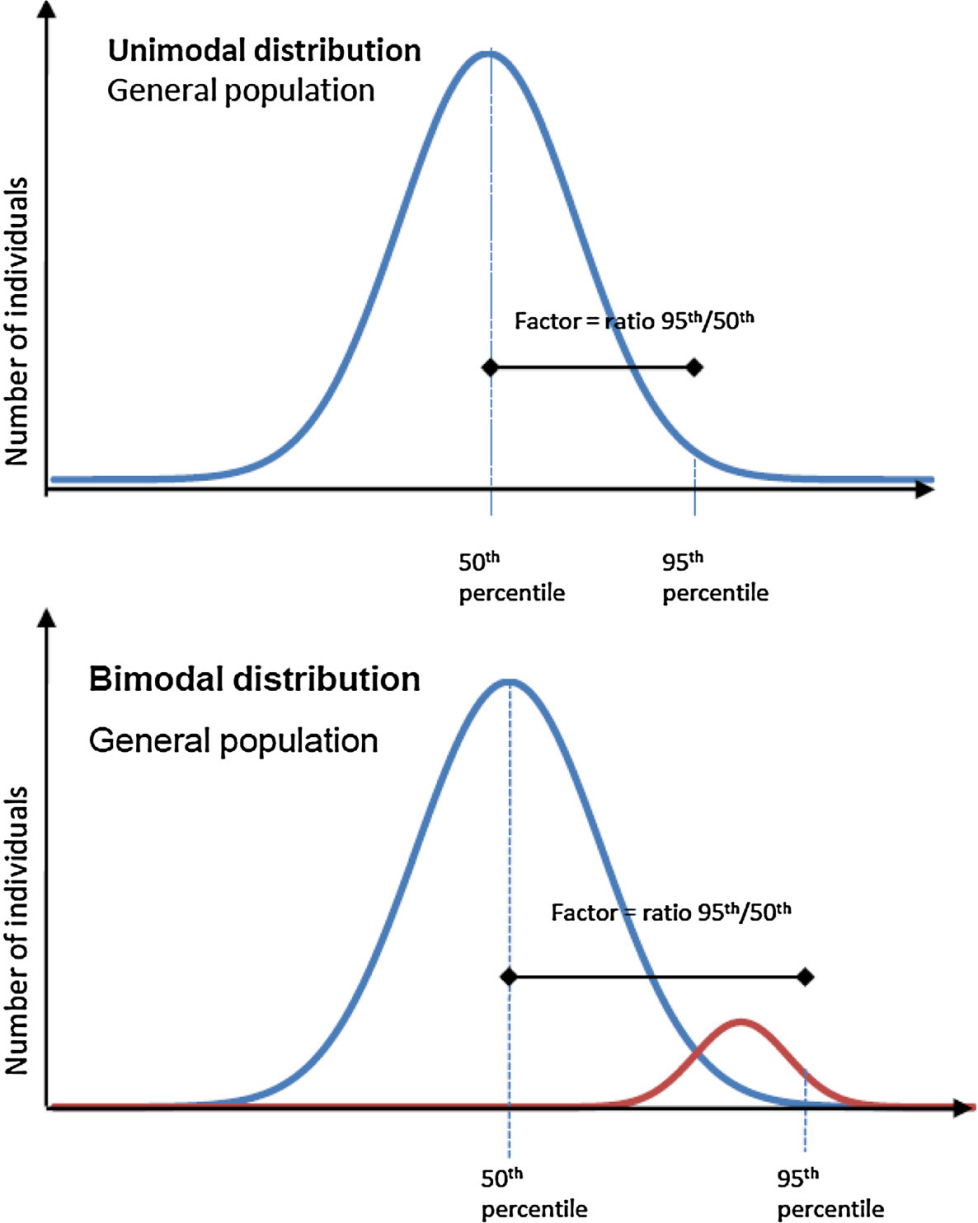
Derivation of intraspecies uncertainty factor from a unimodal parameter or from a bimodal parameter.

The susceptibility of specific population subgroups to the toxic effects of certain chemicals potentially gives rise to multimodal distributions and this contradicts the assumption of unimodality made in the analyses summarised above. The aim is then to protect a reasonable proportion of this sensitive subgroup, and the intraspecies variability factor ought to extend the median for the general population to the percentile of the sensitive subgroup to be protected (see Figure 2).

#### Gender differences

The physiological distinctions between men and women in terms of body weight, tidal volume, water and fat content are relatively small when normalised according to body weight [48]. Nonetheless, differences in lean body mass, percent body fat and to some extent plasma protein concentrations result in variations in volumes of distribution, observed to be consistent with the hydrophilicity or lipophilicity of compounds and gender-dimorphisms in fat content and lean body mass [83]. Gastric pH can influence the absorption of weak acids and bases ingested. Women secrete less gastric acid and have a shorter gastric emptying time. Gastric emptying time is also influenced by sex hormones, particularly during pregnancy and following use of pharmaceutical hormones [83]. Gender disparity has been observed in some Phase I metabolic P450 isoenzymes such as CYP1A2, CYP3A4, CYP2D6, CYP2C19 and is thought to arise from differences in sex hormones, body weight, body water content and diet [61]. Pregnancy influences many physiological parameters such as cardiac output that will in turn influence elimination processes such as the glomerular filtration rate. Lactational excretion of lipophilic compounds is a major gender-specific elimination route, as well as a route of exposure for neonates [61]. Krasovskii [66] reports that the toxicity of 149 substances differed by 2- to 3-fold between male and female rats. Greater toxicodynamic differences are expected to result if the mode-of-action of a substance affects sex-specific differences such as the regulation of hormones or specific damage to the sex organs [3]. Such differences are generally addressed by the design of experimental studies rather than multiplication by a factor.

#### Genetic polymorphisms

Calabrese’s [84] early attempt to characterise human inter-individual variability suggested that genetically determined biochemical differences could result in variability spanning several orders of magnitude. Polymorphisms appear to be more frequent in genes encoding xenobiotic metabolising enzymes than in other genes. It is hypothesised that this may be due to the fact that the former evolved as an adaptive response to the environment. This in turn results in sometimes large ethnic differences in the distribution of specific genotypes [4]. Accordingly, most of the research in this area has focused on polymorphic enzymes involved in xenobiotic metabolism but it is increasingly recognised that genes involved in host (toxicodynamic) responses to chemical stressors can also be polymorphic.

Dorne et al. [80] derived pathway-specific uncertainty factors including some polymorphic metabolic pathways and found that inter-individual variability factors of up to 26 (CYP2C19), 52 (CYP2D6) and 5.2 (*N*-acetyltransferases) would be necessary to protect 99% of poor metabolisers and slow acetylators. The frequency of specific polymorphisms differs between ethnic groups and this led Dorne to assign different pathway-specific uncertainty factors in different ethnic groups [80]. Recent reviews of polymorphisms in metabolism genes and host defence enzymes have been carried out [85,86]. For six phase I and phase II enzymes, information about the influence of the polymorphisms on enzyme function was incorporated with genotype frequency in different ethnic populations to generate distributions of phenotypes. The ratios of medians of all data to the 1st or 99th percentile of the sensitive subgroup generally exceeded the default sub-factor for inter-individual variability of 3.2 with values reaching 90-fold [85].

Moreover, Dorne and Papadopoulos [87] reviewed 200 studies of toxicokinetic interactions such as enzyme induction or inhibition for therapeutic doses of major probe substrates of polymorphic CYPs. The authors’ conclusion was that the default intraspecies subfactor for toxicokinetic differences did not cater for toxicokinetic interactions.

It should nonetheless be noted that these differences in enzymatic activity will not necessarily be reflected in internal doses due to compensating metabolic pathways related to overlapping substrate specificity and other toxicokinetic parameters. PBPK modelling is required to predict the variability of the resulting internal doses. Applied to warfarin and parathion, this approach yielded a 95th percentile to median ratio of 3.4 for parathion and a 99th percentile to median ratio of 26 for warfarin [88], implying uncertainty factors exceeding the default subfactor of 3.2 for inter-individual variabibility in toxicokinetics. However, it is obvious that this approach requires simplifying assumptions in relation to the relative contribution of multiple enzymatic systems to metabolism and the extent of inhibition or induction of these pathways due to co-exposures and other environmental factors [89], and accordingly the outcome of uncertainty factor estimations should be regarded with caution.

#### Differences in health-impaired and elderly people

Ageing affects the efficiency of metabolism and the elimination of xenobiotics. In principle, this could result in a higher sensitivity of elderly people to substances. Particularly, decreases in circulatory efficiency may conceivably compromise the effectiveness with which a compound is distributed in the body. Elderly people are also more likely to be affected by chronic health impairment and excess weight. It is well established that disease can affect the disposition of xenobiotics. Chronic Obstructive Pulmonary Disease is associated with alterations of cardiac output. Heart, kidney and liver disease will expectedly also influence the elimination of harmful substances [90]. Obesity is accompanied by increased blood volume and cardiac output. There is a correlation between obesity and type II diabetes, itself associated with reduced glomerular filtration rates. Moreover, sensitivity to harmful substances is expected to be modified not only when metabolic processes are altered but also when the target organ of the toxic effect is affected by disease [3]. Furthermore there is a possibility of toxicokinetic interaction between environmental contaminants and therapeutic drugs administered to health-impaired individuals. Ginsberg et al. [91] reviewed the literature on therapeutic drug clearance in elderly individuals and found that the half-life of drugs metabolised by hepatic CYP enzymes or via renal elimination is typically 50-75% longer in individuals over 65 years of age compared to younger adults. Thompson et al. [90] compiled a database of physiological parameter values reported in the peer-reviewed literature for healthy and health-impaired elderly (65 years of age or older) freely available from the USEPA website (http://cfpub.epa.gov/ncea/cfm/recordisplay.cfm?deid=201924). Meanwhile, elderly people may also exhibit higher toxicodynamic sensitivity and therefore an intensification of the adverse response to foreign substances [3]. Skowronski and Abdel-Rahman [92] compared toxicokinetic and toxicodynamic differences between healthy adults and geriatrics for six pharmaceuticals and found composite factors were all below the default value of 10 for inter-individual variation. Naumann et al. [42] compiled toxicokinetic and toxicodynamic data on drugs from five different therapeutic classes and calculated adjustment ratios for various sensitive subgroups including the health-impaired and the elderly. Their data show that chemical-specific adjustment factors can be both smaller or greater than default sub-factors, but tend to exceed these default values more often in the health-impaired than the healthy elderly. Dorne et al. [80] calculated pathway-specific uncertainty factors for the elderly subgroup and demonstrated that the toxicokinetic intraspecies subfactor of 3.2 is too small to account for the observed variability. A distributional analysis of the more recent and extensive databases now available is warranted to better characterise the protection afforded to elderly and /or health-impaired people by the default factor.

#### Children, infants and neonates

The sensitivity of children relative to adults has been the subject of some debate in the United States where the protection of children’s health was specifically included in the 1996 Food Quality and Protection Act (U.S. Food and Drug Administration 1996). The issue under discussion was whether the protection of children requires an additional uncertainty factor of 10. An important point to consider is whether young animals were exposed in the study from which a safe level is derived. This is not the case in routine validated experimental protocols for chronic or sub-chronic toxicity. When animals are exposed during critical periods of development such as the prenatal or neonatal period, the ability to predict their susceptibility depends on the adequacy of the animal model. Qualitative comparisons of the maturation of metabolic processes in the rat and the human have been published [78]. Similarly to humans, animal neonates show slower clearance and longer chemical half-lives than adult animals but there are also differences in the rate of maturation of various Phase I or Phase II enzymatic processes and renal function between species. This can equally be said of fetal exposure and cross-species comparisons are typically equally lacking [93]. Dorne et al. [80] calculated some pathway-specific uncertainty factors for children and neonates. All pathway-specific uncertainty factors derived for neonates were above the default value for the intraspecies toxicokinetic subfactor of 3.2 [80]. There are also suggestions that obesity may lead to bimodal distributions of body weight for a given age group of children [94].

Developmental effects that can result from exposures during critical windows of development cannot be predicted, let alone extrapolated, from toxicity observed in adult animals. Nonetheless, children are not generally systematically more sensitive than adults to chemical exposures. Charnley and Putzrath [95] updated a summary of studies of the effects of age on chemically induced carcinogenesis in rodents published in the report of the American National Research Council ‘Pesticides in the diets of Infants and Children’ [96]. Their analysis found that younger animals were more susceptible in 37% of studies whereas older animals were more susceptible in 53% of studies. The biological basis for the sensitivity of children and toxicokinetic differences between children and adults has been comprehensively reviewed [93,97]. The maturation of metabolic processes is of particular interest and most of these processes are reasonably mature by 6 months of age and completely functional by 1 year. A number of studies have investigated the safety afforded to children by the default value for the intraspecies factor either by considering ratios between young and adult animals or those of toxicokinetic and to a lesser extent toxicodynamic parameters between human adults and children. The results of those studies are summarised in Table 5 and suggest that the neonatal period is particularly sensitive. Several studies indicate that default values for the intraspecies variation factor or its toxicokinetic component would be too small for about 30% of chemicals in the first two months of life. This proportion increases to 70% when considering the first week of premature babies.

**Table 5:** Quantitative evaluation on intraspecies differences between adult and the young

**Substances dataset**	**Dose metric**	**Stage of development**	**Results**	**Reference**
*Animal data*				
238 chemicals	LD50	Adult and newborn mammals	Median LD50_Adult_/LD50_Newborn_ = **2.6**	[70]
			14% of ratios exceeded 10	
18 industrial chemicals	pNOAEL	Young and newborn rats (postnatal days 4 to 21)	NOAEL_Young_/NOAEL_Newborn_ < 5 for 17 of 18 chemicals	[98]
*Human data*				
15 anticancer drugs	MTD	Adults and children	1.3 < Mean MTD_Child_/MTD_Adult_ <4.1	[99], ratio of equivalent doses in mg/kg calculated by Dourson et al. [100]
24 drugs	Hepatic clearance (Cl) or half-life (HL)	Adults and newborns	Cl or HL_Newborn_/ Cl or HL_Adult_ > 3.2 for 33% of drugs	[101], ratios calculated by Dourson et al. [100]
22 substances	Toxicokinetic parameters	Adults and children and/or newborns	3.2 subfactor for toxicokinetics intraspecies differences adjusts the adult parameter to that of the infant or child for 91% of substances.	[78] as calculated by Dourson et al. [100]
313 substances (mostly pharmaceuticals)	LD50	Adults and children	14% of LD50_Adult_/LD50_Child_ > 10	[102]
6 drugs	Toxicokinetic parameters (one toxicodynamic)	Adult and children	Ratio of mean adult parameter to lower 95% value of children varied between 0.6-3.7.	[92]
			Composite ratios all below 10	
44 drugs	Half-life	Adult and children in different age groups	Proportion of children whose half-life exceed 3.2-fold the adult mean value;	[94]
			0–1 week premature: 70%	
			0–1 week full term: 26%	
			1 week- 2 months: 27%	
			2 months-18 years: 0%	

### Multiplication of subfactors

On the premise that subfactors are conservative worst-case scenarios, it has been argued that their multiplication results in overly conservative estimates of safe levels for humans [103]. Probabilistic alternatives to the deterministic default factor approach were proposed to help quantify the propagation of uncertainty and level of conservatism. Rather than using single default values, distributions of ratios allow statements about non-observed combinations and therefore are used in extrapolating from animals to healthy adult and then to sensitive individuals. The method requires an accurate characterisation of the distribution of each assessment subfactor and of possible correlations between them. It is generally assumed that all factors are independent and that this premise is sufficiently conservative. As we have stressed earlier, these distributions assume comparability between different types of dose levels, acute and chronic toxicity as well as endpoints. These assumptions have not been subjected to scientific scrutiny. Rather, they are pragmatic approaches that make use of the best available data. The simplest probabilistic approach is by using Monte Carlo simulation techniques [49,104]. However, by using hypothetical case studies, Carlson-Lynch et al. [105] demonstrated that the choice of distribution for the uncertainty factors can yield quantitatively different results and qualitatively different interpretations. Probabilistic approaches have also been used to assess the level of conservativeness of the default 100 uncertainty factor and results of such analysis are shown in. The results of the various approaches vary, predicting that the default uncertainty factor of 100 would be exceeded for less than 5% to nearly 40% of chemicals when derived from experimental data in rodents. The choice of distribution for the subfactors has therefore a great influence. A critical evaluation of these choices should consider whether the distribution was based on data as opposed to a theoretical distribution, whether these data were derived from humans or animals only, and whether adjustment for allometric scaling was included. The only analysis that would meet these requirements is that of [106] for which we calculated that the default uncertainty factor of 100 would be exceeded by 15-20% of chemicals if a safe level for human were to be extrapolated from experimental data in the rat (Table 6). When considering that sensitive subgroups and multimodal distributions of inter-individual sensitivity have not been taken into account in those analyses, an unwavering belief in the undue conservativeness of the default uncertainty factor appears ill-founded Table 6.

**Table 6:** Probabilistic multiplication of subfactors

**Reference**	**Database used**	**Distribution parameters**	**Results**
Sheehan et al. 1990 [70]			
Interspecies variation	Ratios of tumour incidences (TD50s) for 190 chemicals in mice and rats	Median = 2.6	
Intraspecies variation	Ratios of acute lethality (LD50s) for adult and newborn mammals for 238 chemicals	Median = 2.4	
Overall assessment			Values exceeding 100: 11.8% predicted, 10% observed
Baird et al. 1996 [107]			
Interspecies variation	69 pesticides tested in different animal species, allometrically adjusted for body surface (Dourson et al. 1992)	Median = AS^1^	
		GSD = 5	
Intraspecies variation	Probit dose–response slopes from 490 acute lethality experiments using rats [75], assuming two different levels of protection;	Basic approach:	
		Median = 2.7	
		GSD = 2.3	
	Basic approach: 1/100,000	Alternative approach:	
	Alternative approach: 1/1,000	Median = 5.3	
		GSD = 1.4	
Overall assessment	RfDs or RfCs for 126 compounds with NOAELs from chronic bioassays in IRIS database	Basic approach:	Fraction of RfDs within the lower 5% of distribution of potential threshold values^2^ ;
		Median = AS x 3	All: 56%
		P95 = AS x 50	Mice: 23%
		P99 = AS x 220	Rats: 39%
		Alternative approach:	Dogs: 98%
		Median = AS x 5	
		P95 = AS x 63	
		P99 = AS x 194	
Vermeire et al. 1999 ; Vermeire et al. 2001 [5,49]			
Interspecies variation	184 substances tested in mice, rats and dogs	GM = AS	Factor 12 (4 for allometric scaling x 3 for remaining uncertainty) coincides with 73rd percentile.
		GSD = 4.5	
		P95 = AS x 19	
		P99 = AS x 65	
Intraspecies variation	Theoretical, to be consistent with default factor 10, P99 = 10 [108]	Median = 1 + 3	
		GSD = 1.6	
Overall assessment		GM = AS x 4	Percentile of the default factor 100: 79% (NOAEL in mouse), 88% (NOAEL in rat)^3^
		GSD = 4.7	
		P95 = AS x 53	
Gaylor and Kodell 2000 [109]			
Interspecies variation	Binary aquatic interspecies comparisons from dozens to over 500 agents [72]	Median = 1	
		GSD = 1.66	
Intraspecies variation	Probit dose–response slopes from 490 acute lethality experiments using rats [75] adapted by Dourson and Stara [31]	Median = 1	Default value of 10 corresponds to the 92nd percentile
		GSD = 1.64	
Overall assessment		Median = 1	
		GSD = 2.33	
		P95 = 46	
		P99 = 230	
Schneider et al. 2005 [106]			
Interspecies variation	63 antineoplastic agents in humans and five different animal species [68]	GM = AS x 0.97	
		GSD = 3.45	
		P95 = AS x 6.7	
		P99 = AS x 15	
Intraspecies variation	Human database for predominantly healthy adults developed by Hattis et al. [79]	GM = 3.8	
		GSD = 4.3	
		P95 = 44	
		P99 = 117	
Overall assessment	Our own calculation	GM = AS x 3.7	Proportion of substances for which the default factor 100 would not be exceeded:
		GSD = 5.4	AS based on caloric demand; 76% (mouse), 85% (rat)
		P95 = AS x 82	AS based on surface area; 64% (mouse), 79% (rat)
		P99 = AS x 295	
Hasegawa et al. 2010 [110]			
Interspecies variation	63 antineoplastic agents in humans and five different animal species adapted from [68]	GM = AS	
		GSD = 3.23	
		P95 = 48.2 (mice)	
		P95 = 27.5 (rats)	
Intraspecies variation	Rat young/newborn NOAEL ratios for 18 industrial chemicals [98]	GM = 3	
		GSD = 1.38	
		P95 = 5.09	
Overall assessment		P95 = 155 (mice)	
		P95 = 88.7 (rats)	

References

^1^ Allometric scaling factor according to body surface area (mouse = 14, rat = 6).

^2^ For 231 RfDs including some derived from LOAELs and/or sub-chronic bioassays.

^3^ Allometric scaling factors based on caloric demand were used.

Attempts to give uncertainty factors a probabilistic interpretation in terms of response ratios of random chemicals have been argued by some to be ill-conditioned, and Cooke [111] proposed alternative statistical approaches for deriving safe levels such as non-continuous Bayesian belief nets. This would result in new chemicals being assessed on the basis of previous assessments for existing chemicals. Goble and Hattis [50] pointed out that past choices need to be reassessed and potentially revised in view of the level of risk that is considered acceptable to the general public. In accord with these authors, our review demonstrates that the most serious challenge of probabilistic approaches is the representativeness of the distributions from which samples are drawn. For obvious ethical reasons, human studies of the pharmacodynamics or kinetics of chemicals do not include vulnerable populations. The same argument can be used in relation to the representativeness of substances investigated compared to the universe of chemicals the general population is exposed to. However, it is unknown whether the bias introduced by the sets of chemicals in the various databases leads to an underestimation or an overestimation of risks.

### Sufficient protection against mixture effects?

There has been much progress in the thinking of regulatory bodies about combination effects and its implications for the traditional risk assessment paradigm. One important achievement is the acknowledgement by key European scientific committees that NOAELs cannot be equated with zero effect levels (SCHENIR VKM). However, it appears that the implications of this recognition for cumulative risk assessment are not fully appreciated. With an emphasis on human health effects, the EU Scientific Committees stated: “…, if the intended level of protection is achieved for each individual substance, the level of concern for mixtures of dissimilarly acting substances should be assumed as negligible.” This statement is open to interpretation, but seems to suggest that ADIs and similar health-based guidance values, together with the corresponding UFs, also protect against mixture effects.

We are now in a position to examine the two tacit assumptions that underlie this statement, namely that ADI or similar health-based guidance values generally represent zero effect levels, and that the diversity of chemicals which make up human exposures act in strictly independent ways, according to the stochastic principles of the mixture concept of independent action. Only if both assumptions can be shown to be realistic, are combination effects not to be expected. In such a situation, the protection achieved for single substances by using UFs for ADI setting also safeguards against mixture effects.

Our examination of the literature has shown that health-based guidance values cannot be demonstrated to represent absolute zero effect levels, as is recognised by the intended level of protection enunciated in the ‘Straw Man’ proposal. Even if we assume that the mixture assessment concept of independent action is applicable to the multitude of chemicals relevant to human exposures, certain risks may arise from small effects associated with single chemicals which might cumulate according to stochastic principles. For example, independent action assumes that 100 chemicals each associated with an effect of 0.01% will produce a combination effect of 0.97%.

Considering the diversity of chemicals human beings are exposed to, it is intuitively appealing to assume that independent action, with its assumptions about different mechanisms and modes of action, should be generally applicable for an assessment of mixture effects. However, the applicability of independent action to mammalian organisms and cells has yet to be demonstrated. There is no case described in the literature, where independent action produced valid descriptions of combinations of chemicals that produce shared toxic effects by different mechanisms [12]. All the available evidence points in the opposite direction: independent action consistently underestimated the combined effects of various chemicals with different mechanisms. These findings have led many international bodies to recommend the use of dose addition as the default concept (IPCS 2009). This was also supported by the Scientific Committees.

## Discussion

The evidence analysed in the preceding sections shows that default uncertainty factors do not represent worst-case scenarios and were not intended to do so. The scheme originally proposed by Renwick [37] was suggested to represent an adequate scenario in most but not all cases. Two conclusions can be drawn from our analysis. The first is that due to intractable ethical issues about testing in humans, the database on which the adequacy of the uncertainty factors can be judged is necessarily poor. This introduces uncertainty and whether this may lead to uncertainty factors that overestimate or underestimate the risk to humans can only be speculated upon but not scientifically proven. Secondly, the data available does not support an unwavering belief in the purported conservativeness of the overall default uncertainty factor of 100.

In a regulatory context, where the replacement of default uncertainty factors by chemical- or pathway-specific factors is encouraged, default uncertainty factors that represent reasonable worst-case scenarios would provide an incentive to generate better (chemical- or pathway-specific) data. We believe this is not currently the case and a revision of the current default uncertainty factors deserves consideration.

The level of conservatism (or otherwise) of uncertainty factors can only be considered in relation to a defined level of protection. Absolute zero-effect thresholds for the human population cannot be empirically determined. There is therefore a need to decide what constitutes an appreciable risk or the desired level of protection. This decision entails the consideration of intractable and considerable uncertainty associated with the extrapolations from observed effects in experimental animals to small effect magnitudes, often over several orders of magnitude. It therefore follows that statements about the conservativeness of uncertainty factors, the safety of chemicals or mixtures invariably withhold hidden beliefs and value judgments. These need to be explicitly stated for an intelligent debate about the desired values attributed to default uncertainty factors to take place.

This has important implications for mixture risk assessment, particularly in the case where independent action is presumed. If absolute zero-effect threshold cannot be demonstrated in the human population, we do not concur that the combined effect(s) of chemicals with independent modes-of-action below levels derived to be ‘safe’ for single substances should be assumed to be negligible. The assessment of such potential effects requires knowledge of the exposure to all chemicals during an individual’s lifetime, (now often referred to as the exposome) and this knowledge is at present generally lacking. This knowledge gap was recognised by the Scientific Committees in their opinion and by the European Commission in its recent communication. We further argue that this incomplete picture would impede the prioritisation of mixtures of concern as recommended by the Commission. We therefore contend that precautionary regulation should provide an incentive to generate better data and recommend that a pragmatic approach is taken to mixture risk assessment. In addition to detailed assessments of mixtures identified as ‘of concern’, building additional safety in single chemical risk assessment, whether in the form of an additional uncertainty factor, larger margins of safety or exposure or similar approaches, should be considered by the ad hoc working group to be established by the European Commission as a default for all substances until better knowledge of other relevant exposures and information about the mode-of-action can be ascertained.

## Conclusions

The present review demonstrates unequivocally that assuming that default uncertainty factors are overly conservative worst-case scenarios that could account both for the lack of statistical power in animal experiments and protect against potential mixture effects are ill-founded. It is high time such urban myths ceased being cited as gospel.

## Abbreviations

ADI: Acceptable daily intake; AhR: Arylhydrocarbon receptor; BMDL: Benchmark dose level; CYP: Cytochrome 450; ECETOC: European centre for ecotoxicology and toxicology of chemicals; ECHA: European chemical agency; EPA: Environmental protection agency; EUROTOX: European committee for the protection of the population against the hazards of chronic toxicity; FAO: Food and Agriculture Organization; GSD: Geometric standard deviation; IPCS: International programme on chemical safety; JEFCA: Joint expert committee on food additives; JMPR: Joint meeting on pesticides residues; KEMI: Swedish national chemicals inspectorate; LD50: Median lethal dose; LOAEL: Lowest observable adverse effect level; MTD: Maximum tolerated dose; NOAEL: No observed adverse effect level; PBPK: Pharmacologically-based pharmacokinetic modelling; PCB: Polychlorinated biphenyls; REACH: Registration, Evaluation, authorisation and restriction of chemicals (EC 1907/2006); RfD: Reference dose; SCCS: Scientific committee on consumer safety; SCENIHR: Scientific committee on emerging and newly identified health risk; SCHER: Scientific committee on health and environmental risks; TCDD: 2,3,7,8-tetrachlorodibenzo-p-dioxin; TDI: Tolerable daily intake; WHO: World Health Organization.

## Competing interests

The authors declare that they have no competing interest.

## Authors’ contributions

OVM designed and carried out the study and drafted the manuscript. MS participated in the conception of the study and provided statistical advice and expertise. AK participated in the conception of the study, provided expertise in mixture toxicology, risk assessment and regulation and drafted the relevant sections of the manuscript. All authors read and approved the final manuscript.
